# Role of gender on academic performance based on different parameters: Data from secondary school education

**DOI:** 10.1016/j.dib.2020.105257

**Published:** 2020-02-10

**Authors:** S. Ranjeeth, T.P. Latchoumi, P.Victer Paul

**Affiliations:** a Department of Computer Science and Engineering, VFSTR (Deemed to be University), Guntur, AP, India; b Department of Computer Science and Engineering, Indian Institute of Information Technology Kottayam, Kerala, India

**Keywords:** Learning analytics, Higher secondary, Education data mining, Academic performance

## Abstract

This data article represents academic performances and personal habits of higher secondary school students in select high school education institutions located at Guntur, Andhra Pradesh, India for the academic year of 2017–18. The dataset of 1116 records, each with 9 parameters and the parameters are mother and father education, the impact of advisor, time spent on study after school, time spent on sports, time spent with mobile per day, the impact of health problems, goal, time spent on yoga or physical exercise. We present descriptive statistics showing mean, median, mode, maximum, minimum, range, standard deviation and variance in the performances of these students and a bar chart representation of the total number of female and male students' Grade Point Average (GPA) with 9 parameters individually. The primary aim of this clause is providing a comparative analysis of female and male students in GPA scoring based on 9 parameters.

**Table:** Specifications Table

Subject	Secondary school Education
Specific subject area	Learning analytics
Type of data	Tables, figures, excel file, graphs
How data were acquired	Data selected from secondary education institutes led a survey through a questionnaire in the academic year of 2018
Data format	Raw, analyzed
Parameters for data collection	Secondary students with complete records were included and considering some parameters in this study
Description of data collection	This data is collected through a survey in the form of a questionnaire from secondary education institutes in Guntur, India.
Data source location	Information is collected from secondary education institutes in Guntur (Latitude 16.3067° N, Longitude 80.4365° E), Andhra Pradesh, India
Data accessibility	Data are with this article
Related research article	**Author's name** Temitope M. John, Joke A. Badejo, Segun I. Popoola **Title:** The role of gender on academic performance in STEM-related disciplines: Data from a tertiary institution. **Journal: Data In Brief** DOI: https://doi.org/10.1016/j.dib.2018.03.052

**Table:** 

**Value of the Data** • The parameters that greatly influence the economic development of the country are taken into consideration to measure student's academic performance • The collected information's enhance to the individual student need • Information received will inculcate social awareness, fulfill the requirements of each student towards employability and higher studies • The outcome of this data shows the capability to predict the Student Academic Performance and is highly beneficial to take remedial actions in the present educational system.

## 1 Data description

Education is very important for all and it plays a crucial role in their lives, in this article focused on secondary education (10th class (SSC)) passed out (the academic year 2017–18) students’ data, which is gathered from secondary education institutes through a questionnaire in the contour of a survey in Guntur, Andhra Pradesh, India. Data contains a total of 1116 understudies GPA with 9 different parameters, parameters like mother and father education, impact of advisor, time spent on study after school per day, time spent on sports per day, time spent with mobile per day, the impact of health problems, goal and time spent on yoga or physical workouts [[1], [2], [3], [4], [5]]. The principal objective of this data article is gender-wise performance analysis based on different parameters, female and male students are outperformed in scoring GPA more students are scored between 9.0 and 9.9 .

Descriptive statistics on the number of female and male students’ GPA performance of the academic year 2018 based on 9 different parameters are recorded in Table 1, Table 2, Table 3, Table 4, Table 5, Table 6, Table 7, Table 8, Table 9.

**Table 1:** Descriptive statistics of the number of female and male student's GPA (the academic year 2017–18) with parameter (Mother education).

Mother Education	Gender	Mean	std	Min	25%	50%	75%	Max	Total
None and Below SSC	Female	26.33	28.98	0.00	7.00	17.50	35.50	78.00	158
	Male	50.16	57.70	1.00	11.00	30.5	68.75	153.00	301
SSC	Female	19.83	27.69	1.00	2.50	17.50	35.50	73.00	119
	Male	29.33	34.45	0.00	6.25	20.50	35.50	93.00	176
Intermediate	Female	12.00	14.09	0.00	2.25	8.50	14.75	38.00	72
	Male	15.66	18.29	0.00	2.75	11.00	20.00	49.00	94
Graduate	Female	9.50	10.15	0.00	1.25	7.50	14.50	26.00	57
	Male	13.83	15.18	0.00	4.00	11.00	15.75	42.00	83
PG and More	Female	4.00	4.97	0.00	0.25	3.00	5.00	13.00	24
	Male	5.33	8.28	0.00	1.25	2.50	3.75	22.00	32

**Table 2:** Descriptive statistics of the number of female and male student's GPA (the academic year 2017–18) with parameter (Father education).

Father education	Gender	Mean	std	Min	25%	50%	75%	Max	Total
None and Below SSC	Female	22.66	25.68	0.00	5.50	13.00	33.25	67.00	136
	Male	39.00	46.35	1.00	5.75	23.50	54.75	121.00	234
SSC	Female	13.00	17.06	0.00	2.75	7.50	13.75	46.00	78
	Male	21.83	24.82	0.00	7.00	13.00	28.00	67.00	131
Intermediate	Female	14.66	16.37	1.00	2.75	10.00	19.50	44.00	88
	Male	21.50	24.90	0.00	5.00	14.50	27.00	67.00	129
Graduate	Female	15.50	18.99	0.00	2.00	12.00	17.50	51.00	93
	Male	21.16	23.44	0.00	5.50	15.50	26.25	64.00	127
PG and More	Female	5.83	7.70	0.00	0.25	3.50	7.50	20.00	35
	Male	10.83	14.68	0.00	3.25	7.00	8.50	40.00	65

**Table 3:** Descriptive statistics of the number of female and male student's GPA (the academic year 2017–18) with parameter (Advisor impact).

Advisor Impact	Gender	Mean	std	Min	25%	50%	75%	Max	Total
Low	Female	2.66	2.06	0.00	1.25	2.50	4.50	5.00	16
	Male	4.83	7.62	0.00	1.00	1.50	4.25	20.00	29
Medium	Female	26.33	31.28	0.00	7.75	2.50	35.00	83.00	158
	Male	42.00	44.97	1.00	7.75	31.50	59.75	119.00	252
High	Female	25.16	32.10	0.00	3.25	18.50	27.00	86.00	151
	Male	41.00	50.76	0.00	11.00	21.50	50.00	136.00	246
Very High	Female	9.83	11.44	0.00	0.25	8.00	15.00	28.00	59
	Male	19.50	21.62	0.00	4.00	17.50	22.00	59.00	117
No Impact	Female	7.66	9.85	0.00	1.25	4.00	9.75	26.00	46
	Male	7.16	9.86	0.00	0.25	3.00	10.25	25.00	43

**Table 4:** Descriptive statistics of the number of female and male student's GPA (the academic year 2017–18) with parameter (time spent on study after school).

Time spent	Gender	Mean	std	Min	25%	50%	75%	Max	Total
No time	Female	11.16	11.82	1.00	3.00	8.50	13.25	33.00	67
	Male	22.66	26.52	0.00	4.00	14.00	32.25	69.00	136
1hr	Female	14.33	15.83	0.00	4.75	10.50	16.25	44.00	86
	Male	25.00	25.61	0.00	7.25	20.00	32.75	70.00	150
2hrs	Female	19.50	23.35	0.00	3.50	11.00	27.50	61.00	117
	Male	25.83	33.43	0.00	4.25	14.50	30.75	89.00	155
More than 2hrs	Female	26.66	34.16	0.00	2.00	18.00	32.50	90.00	160
	Male	40.83	47.94	1.00	11.00	26.00	47.75	131.00	245

**Table 5:** Descriptive statistics of the number of female and male student's GPA (the academic year 2017–18) with parameter (time spent on sports).

Time spent	Gender	Mean	std	Min	25%	50%	75%	Max	Total
No time	Female	33.33	40.05	0.00	3.75	20.50	48.50	103.00	200
	Male	26.66	32.43	0.00	6.25	17.50	30.25	88.00	160
less than 30 mins	Female	20.83	25.38	0.00	6.25	14.50	20.50	70.00	125
	Male	30.16	38.18	0.00	6.75	21.00	30.00	104.00	181
1–2hrs	Female	14.16	16.14	1.00	2.75	10.00	17.25	44.00	85
	Male	42.50	50.05	0.00	7.00	27.00	58.25	132.00	255
More than 2hrs	Female	3.66	4.45	0.00	0.50	3.00	4.00	12.00	22
	Male	15.00	14.89	1.00	4.25	9.00	27.25	35.00	90

**Table 6:** Descriptive statistics of the number of female and male student's GPA (the academic year 2017–18) with parameter (time spent with mobile).

Time spent	Gender	Mean	std	Min	25%	50%	75%	Max	Total
No time	Female	17.50	22.34	0.00	1.75	9.50	24.00	58.00	105
	Male	24.50	29.16	0.00	4.75	16.50	30.50	78.00	147
30 mins	Female	24.00	27.75	0.00	5.25	17.00	29.50	75.00	144
	Male	20.83	24.95	0.00	6.50	11.00	26.00	67.00	125
1hr	Female	11.33	11.39	0.00	3.00	10.00	14.75	31.00	68
	Male	22.00	26.75	0.00	5.25	15.00	24.00	73.00	132
2hrs	Female	9.50	12.19	0.00	1.50	4.00	14.00	31.00	57
	Male	19.83	23.45	0.00	3.75	11.00	28.75	61.00	119
More than 2hrs	Female	9.33	12.27	1.00	1.75	4.50	10.25	33.00	56
	Male	27.16	29.41	0.00	6.75	21.00	34.50	80.00	163

**Table 7:** Descriptive statistics of the number of female and male student's GPA (the academic year 2017–18) with parameter (impact of health problems).

Health problems	Gender	Mean	std	Min	25%	50%	75%	Max	Total
Always	Female	3.00	2.82	0.00	0.50	3.00	4.75	7.00	18
	Male	5.50	4.84	0.00	1.25	6.00	8.50	12.00	33
Most of the time	Female	3.83	3.71	0.00	1.25	3.00	5.50	10.00	23
	Male	7.00	9.20	0.00	2.00	4.00	6.75	25.00	42
Some times	Female	25.10	28.57	1.00	6.50	16.50	31.00	78.00	151
	Male	34.33	37.08	1.00	6.25	24.00	50.75	97.00	206
Rarely	Female	31.33	42.41	0.00	1.00	18.50	39.00	110.00	188
	Male	50.00	64.55	0.00	12.75	30.00	51.00	175.00	300
Never	Female	8.33	8.23	0.00	3.25	6.50	10.50	23.00	50
	Male	17.50	19.55	0.00	4.00	10.50	26.75	50.00	105

**Table 8:** Descriptive statistics of the number of female and male student's GPA (the academic year 2017–18) with parameter (impact of goal).

Goal	Gender	Mean	std	Min	25%	50%	75%	Max	Total
Engineer	Female	38.33	51.06	0.00	3.75	24.00	43.50	135.00	230
	Male	48.66	61.77	0.00	9.75	29.50	55.25	166.00	292
Doctor	Female	3.83	5.19	0.00	1.00	2.00	3.75	14.00	23
	Male	3.66	3.55	0.00	1.25	2.50	6.00	9.00	22
Actor	Female	0.83	0.75	0.00	0.25	1.00	1.00	2.00	05
	Male	3.83	3.65	0.00	1.25	3.50	5.00	10.00	23
Teacher	Female	8.00	7.58	0.00	2.00	7.00	13.50	18.00	48
	Male	1.83	2.31	0.00	0.25	1.00	2.50	6.00	11
Politician	Female	0.83	0.75	0.00	0.25	1.00	1.00	2.00	05
	Male	4.66	6.12	0.00	0.25	1.50	9.50	13.00	28
Any other	Female	19.83	22.85	0	3	12.5	29.5	59	119
	Male	51.66	57.58	1	11.5	37	66.25	156	310

**Table 9:** Descriptive statistics of the number of female and male student's GPA (the academic year 2017–18) with parameter (time spent on yoga).

Yoga Time	Gender	Mean	std	Min	25%	50%	75%	Max	Total
Morning Time	Female	14.00	16.68	0.00	1.50	10.00	18.50	44.00	84
	Male	29.66	30.50	0.00	7.25	22.00	44.25	80.00	178
Evening Time	Female	4.50	4.13	1.00	1.25	3.00	7.00	11.00	27
	Male	9.16	10.45	0.00	2.75	7.00	9.75	29.00	55
In Free time	Female	19.00	23.17	0.00	20.00	14.00	23.75	61.00	114
	Male	28.66	35.43	0.00	6.50	18.50	32.00	96.00	172
Not Interested	Female	34.16	41.40	0.00	8.50	20.50	40.75	112.00	205
	Male	46.83	57.64	1.00	10.00	26.50	58.00	154.00	281

## 2 Experimental design, materials, and methods

Data of female and male secondary education students were collected from the secondary education institutes in the form of a questionnaire and got an official approval letter from secondary educational institutes in India the data contains the final GPA (Grade Point Average) of the academic year 2017–18 with 9 parameters. Figures from 1 to 18 shows the bar chart of the total number of female and male students’ GPA based on nine different parameters that provides in Table 10, Table 11, Table 12, Table 13, Table 14, Table 15, Table 16, Table 17, Table 18, Table 19, Table 20.

**Table 10:** The number of female students’ GPA (the academic year 2017–18) based on mother education.

GPA	None and Below SSC	SSC	Intermediate	Graduate	Pg and More/JOB
10	22	13	11	16	5
9.0 to 9.9	78	73	38	26	13
8.0 to 8.9	40	26	16	10	5
7.0 to 7.9	13	4	6	5	1
6.0 to 6.9	5	2	1	0	0
5.0 to 5.9	0	1	0	0	0
Total	158	119	72	57	24

**Table 11:** The number of male students’ GPA (the academic year 2017–18) based on mother education.

GPA	None and Below SSC	SSC	Intermediate	Graduate	Pg and More/JOB
10	26	19	8	10	1
9.0 to 9.9	153	93	49	42	22
8.0 to 8.9	80	40	22	17	4
7.0 to 7.9	35	22	14	12	2
6.0 to 6.9	6	2	1	2	3
5.0 to 5.9	1	0	0	0	0
Total	301	176	94	83	32

**Table 12:** The number of female students’ GPA (the academic year 2017–18) based on father education.

GPA	None and Below SSC	SSC	Intermediate	Graduate	Pg and More/JOB
10	16	10	15	18	8
9.0 to 9.9	67	46	44	51	20
8.0 to 8.9	39	15	21	16	6
7.0 to 7.9	10	5	5	8	1
6.0 to 6.9	4	2	2	0	0
5.0 to 5.9	0	0	1	0	0
Total	136	78	88	93	35

**Table 13:** The number of male students’ GPA (the academic year 2017–18) based on father education.

GPA	None and Below SSC	SSC	Intermediate	Graduate	Pg and More/JOB
10	20	13	14	10	7
9.0 to 9.9	121	67	67	64	40
8.0 to 8.9	64	33	31	28	7
7.0 to 7.9	27	13	15	21	9
6.0 to 6.9	1	5	2	4	2
5.0 to 5.9	1	0	0	0	0
Total	234	131	129	127	65

**Table 14:** The number of female students’ GPA (the academic year 2017–18) based advisor impact.

GPA	NO IMPACT	LOW	MEDIUM	HIGH	VERY HIGH
6.0 to 6.9	0	0	0	0	0
5.0 to 5.9	1	1	6	1	0
7.0 to 7.9	2	3	13	10	1
10	6	5	14	27	15
8.0 to 8.9	11	2	42	27	15
9.0 to 9.9	26	5	83	86	28
Total	46	16	158	151	59

**Table 15:** The number of male students’ GPA (the academic year 2017–18) based advisor impact.

GPA	NO IMPACT	LOW	MEDIUM	HIGH	VERY HIGH
5.0 to 5.9	0	0	1	0	0
6.0 to 6.9	0	1	4	8	1
10	1	2	19	20	22
7.0 to 7.9	5	1	44	23	13
8.0 to 8.9	12	5	65	59	22
9.0 to 9.9	25	20	119	136	59
Total	43	29	252	246	117

**Table 16:** The number of female students’ GPA (the academic year 2017–18) based time spent on study after school per day.

GPA	no time spent	1 hr	2 hrs	More than 2hrs
10	11	14	14	28
9.0 to 9.9	33	44	61	90
8.0 to 8.9	14	17	32	34
7.0 to 7.9	6	7	8	8
6.0 to 6.9	2	4	2	0
5.0 to 5.9	1	0	0	0
Total	67	86	117	160

**Table 17:** The number of male students’ GPA (the academic year 2017–18) based time spent on study after school per day.

GPA	No time spent	1 hr	2 hrs	More than 2hrs
10	13	17	11	23
9.0 to 9.9	69	70	89	131
8.0 to 8.9	38	36	35	54
7.0 to 7.9	15	23	18	29
6.0 to 6.9	1	4	2	7
5.0 to 5.9	0	0	0	1
Total	136	150	155	245

**Table 18:** The number of female students’ GPA (the academic year 2017–18) based time spent on sports per day.

GPA	No time spent	Less than 30 mins	1–2hrs	More than 2hrs
10	35	16	12	4
9.0 to 9.9	103	70	44	12
8.0 to 8.9	53	22	19	4
7.0 to 7.9	6	13	8	2
6.0 to 6.9	3	4	1	0
5.0 to 5.9	0	0	1	0
Total	200	125	85	22

**Table 19:** The number of male students’ GPA (the academic year 2017–18) based time spent on sports per day.

GPA	No time spent	Less than 30 mins	1–2hrs	More than 2hrs
10	19	24	16	5
9.0 to 9.9	88	104	132	35
8.0 to 8.9	34	32	65	32
7.0 to 7.9	16	18	38	13
6.0 to 6.9	3	3	4	4
5.0 to 5.9	0	0	0	1
Total	160	181	255	90

**Table 20:** The number of female students’ GPA (the academic year 2017–18) based time spent with mobile per day.

GPA	No time spent	30 mins	1hr	2 hrs	More than 2hr
10	15	31	11	5	5
9.0 to 9.9	58	75	31	31	33
8.0 to 8.9	27	25	16	17	12
7.0 to 7.9	4	9	9	3	4
6.0 to 6.9	1	4	1	1	1
5.0 to 5.9	0	0	0	0	1
Total	105	144	68	57	56

**Fig. 1:**
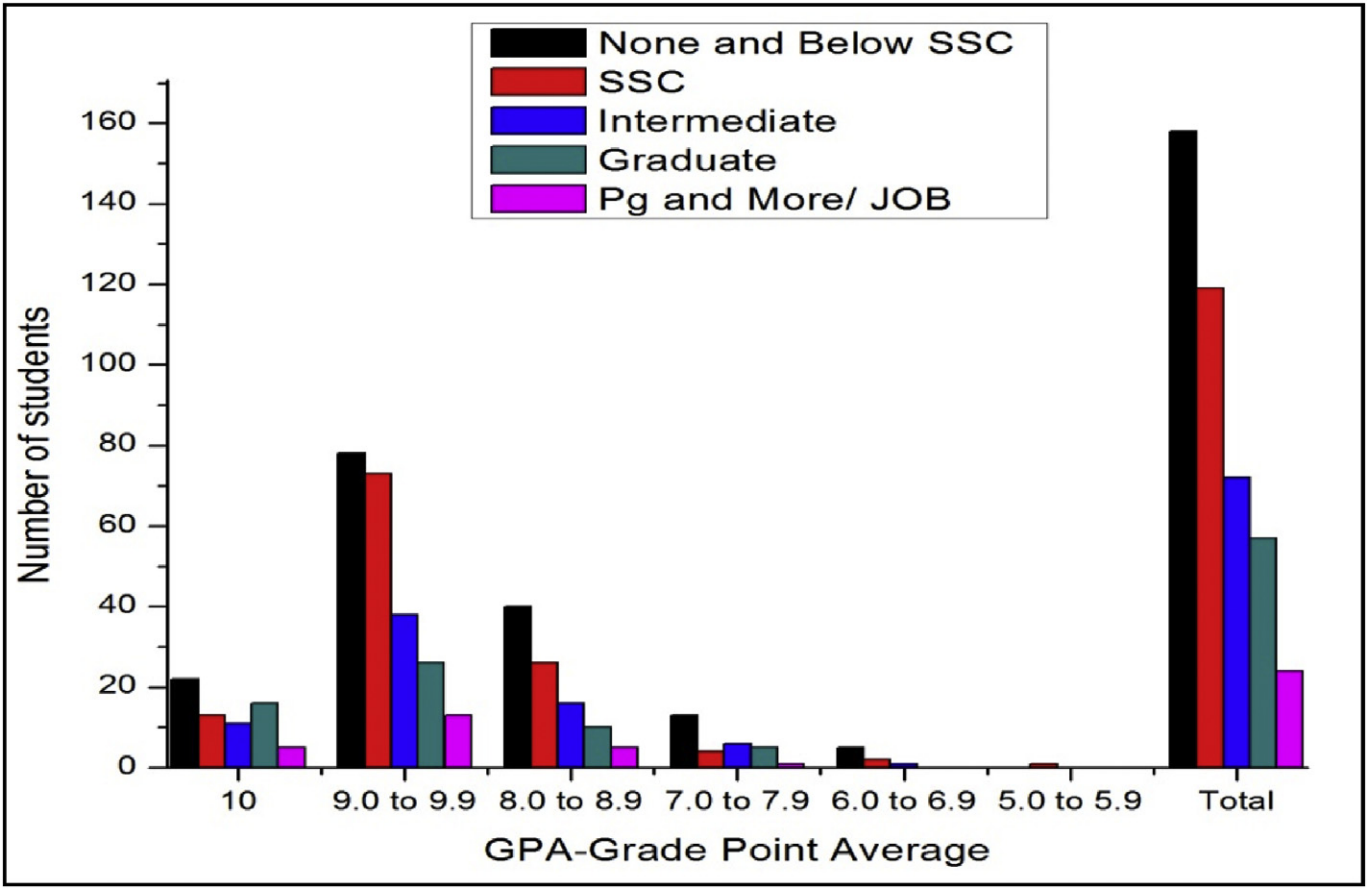
The bar chart shows the number of female students' GPA (the academic year 2017–18) based on mother education.

**Fig. 2:**
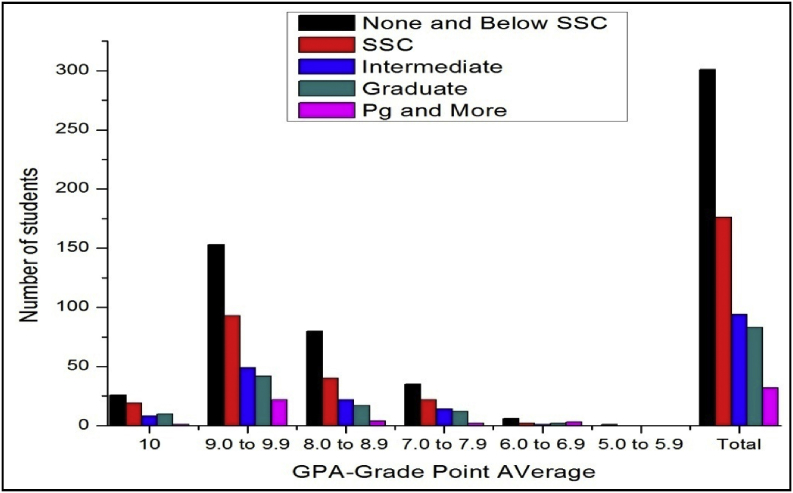
The bar chart shows the number of male student's GPA (the academic year 2017–18) based on mother education.

The total number of female and male students’ GPA based on mother education qualification is shown in Fig. 1, Fig. 2.

**Fig. 3:**
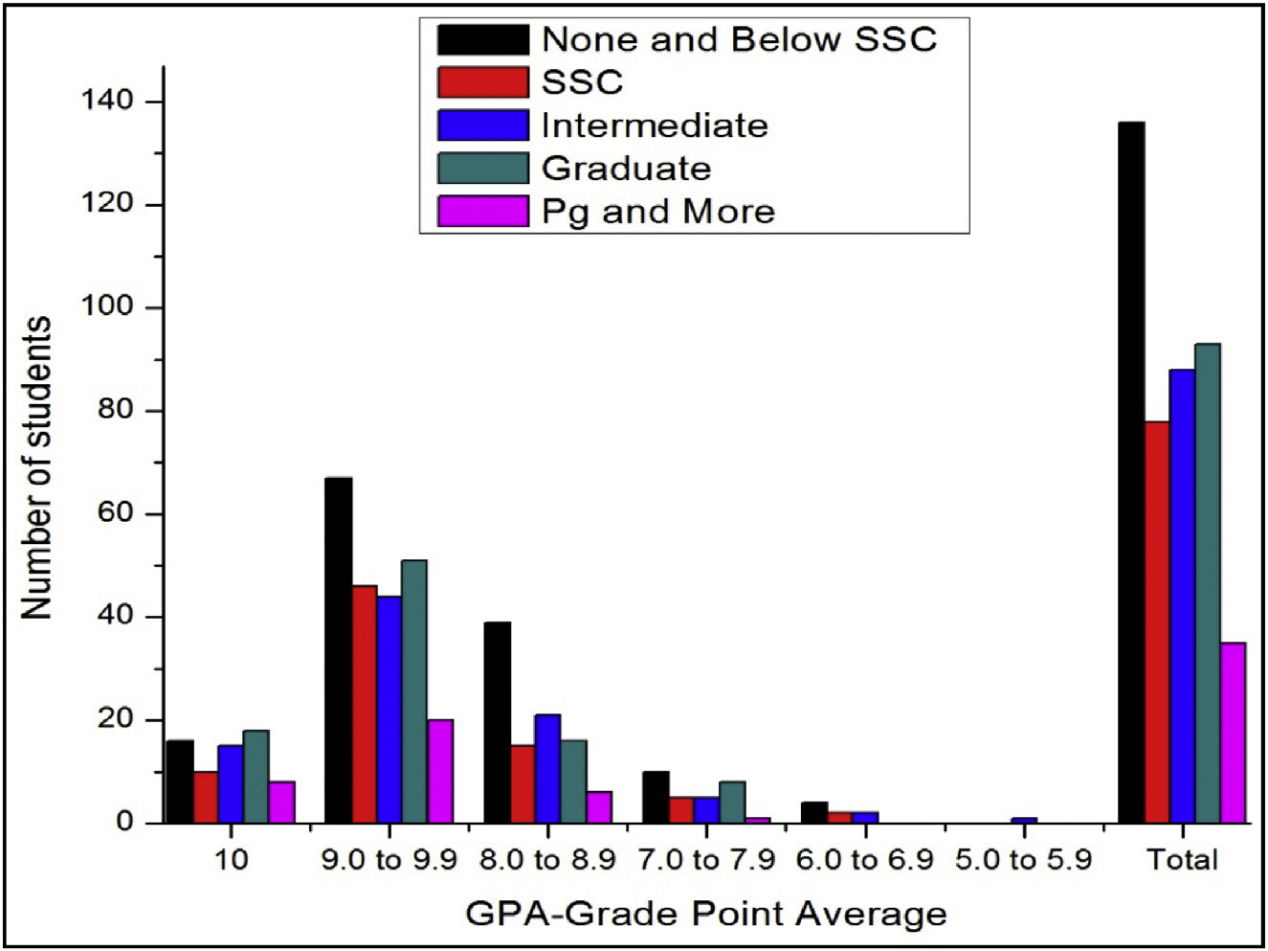
The bar chart shows the number of female students' GPA (the academic year 2017–18) based on father education.

**Fig. 4:**
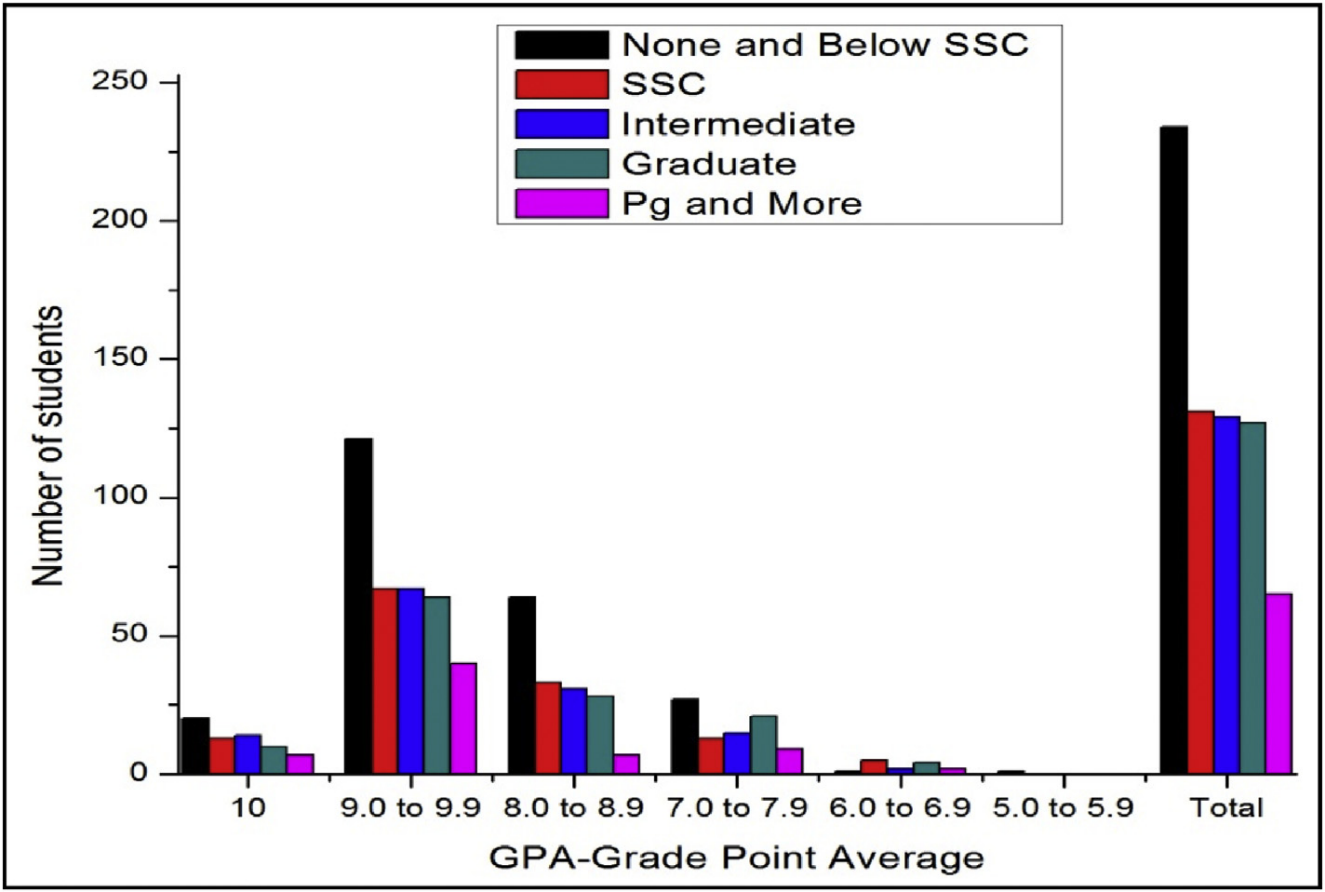
The bar chart shows the number of male students' GPA (the academic year 2017–18) based on father education.

The total number of female and male students’ GPA based on father education qualification is shown in Fig. 3, Fig. 4.

**Fig. 5:**
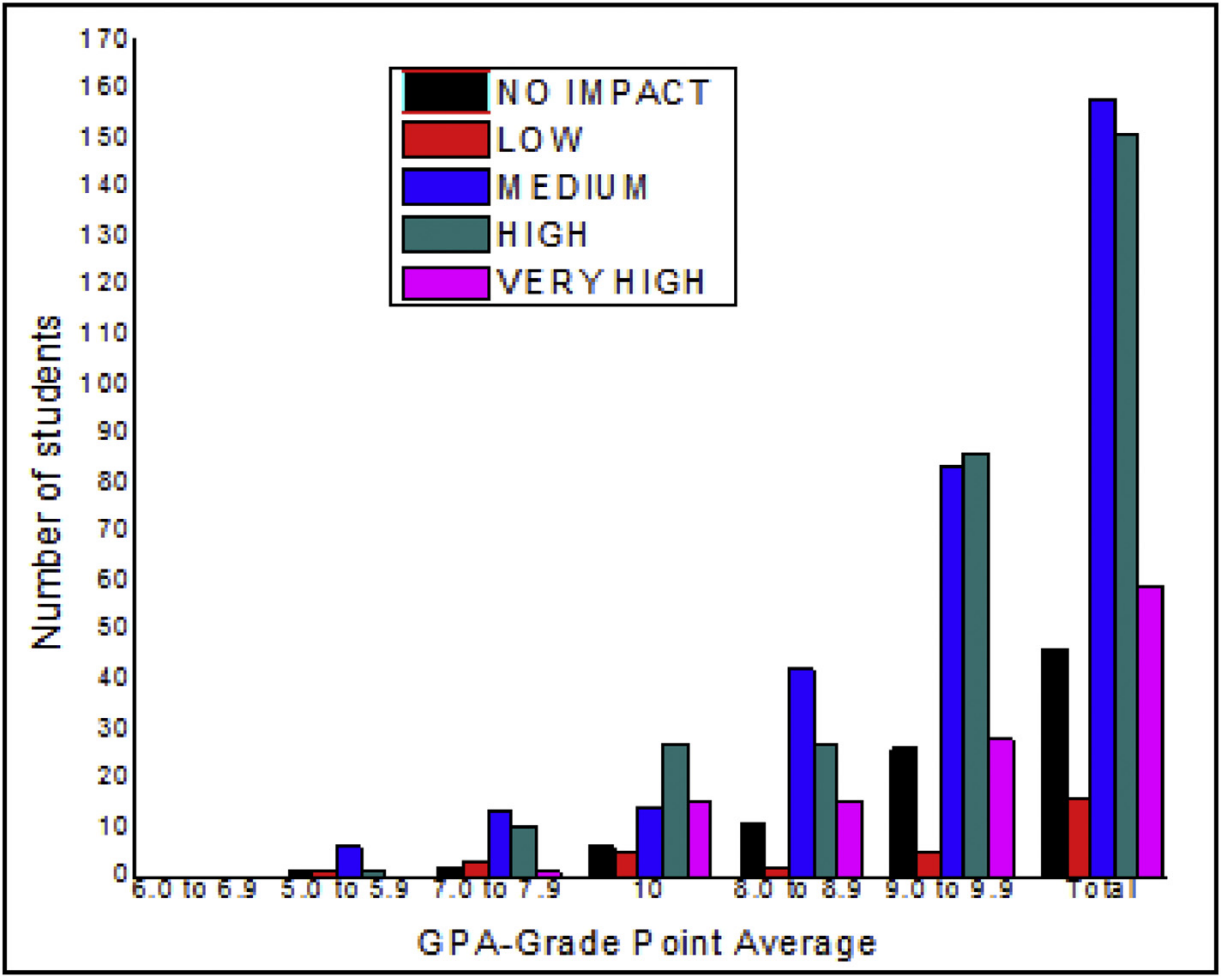
The bar chart shows the number of female students' GPA (the academic year 2017–18) based advisor impact.

**Fig. 6:**
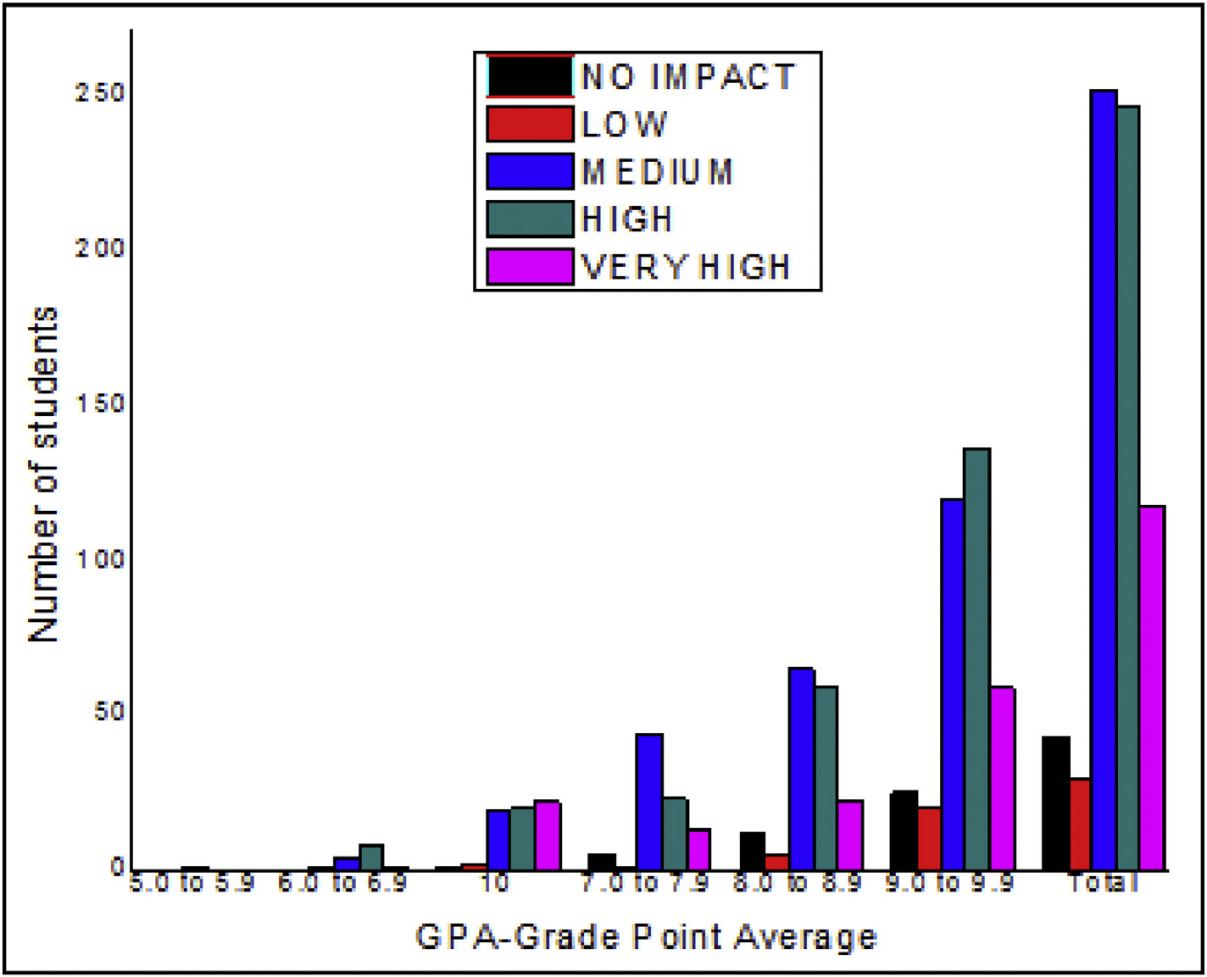
The bar chart shows the number of male students' GPA (the academic year 2017–18) based advisor impact.

The total number of female and male students’ GPA based on advisor impact shown in Fig. 5, Fig. 6.

**Fig. 7:**
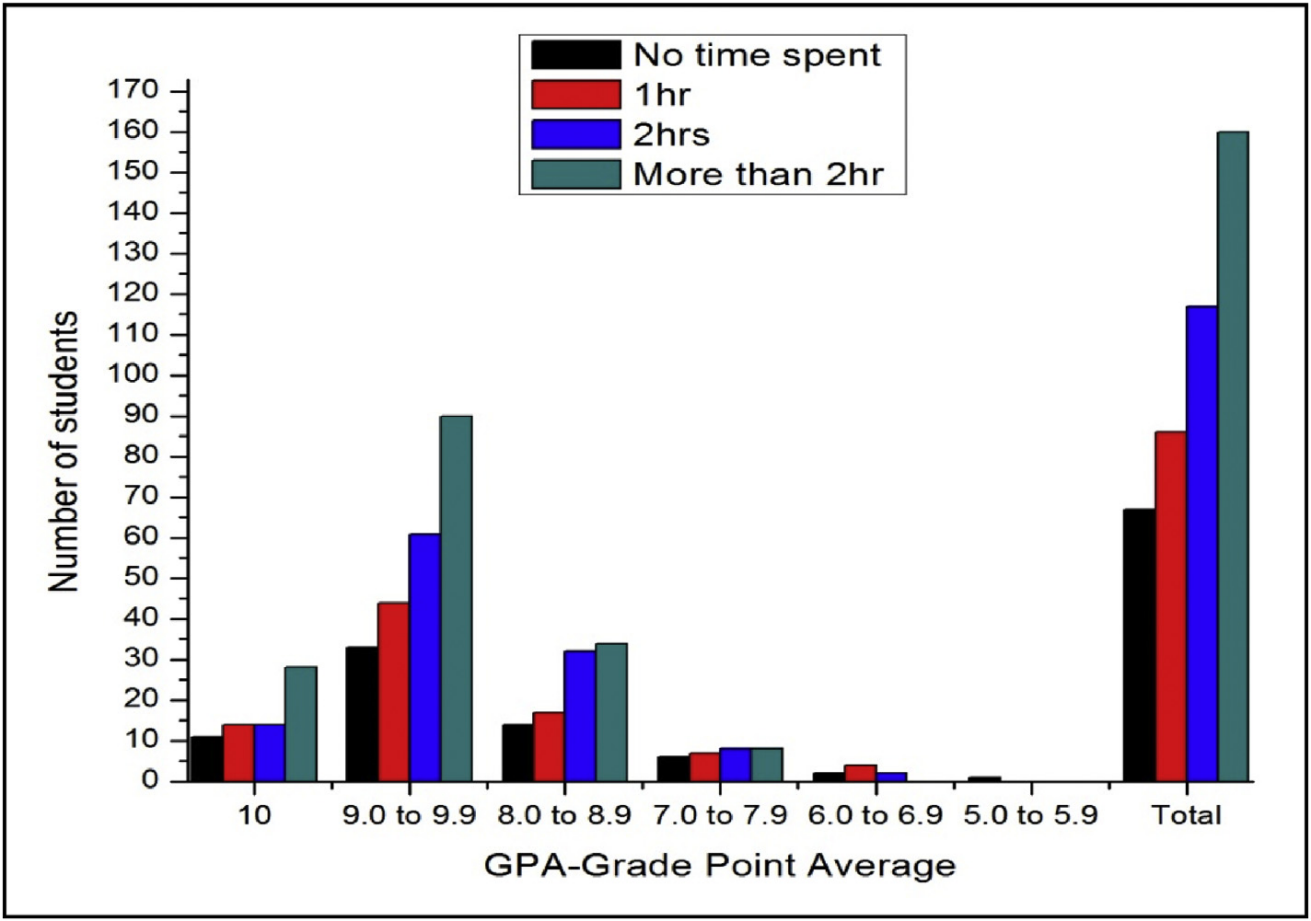
The bar chart shows the number of female students' GPA (the academic year 2017–18) based time spent on study after school per day.

**Fig. 8:**
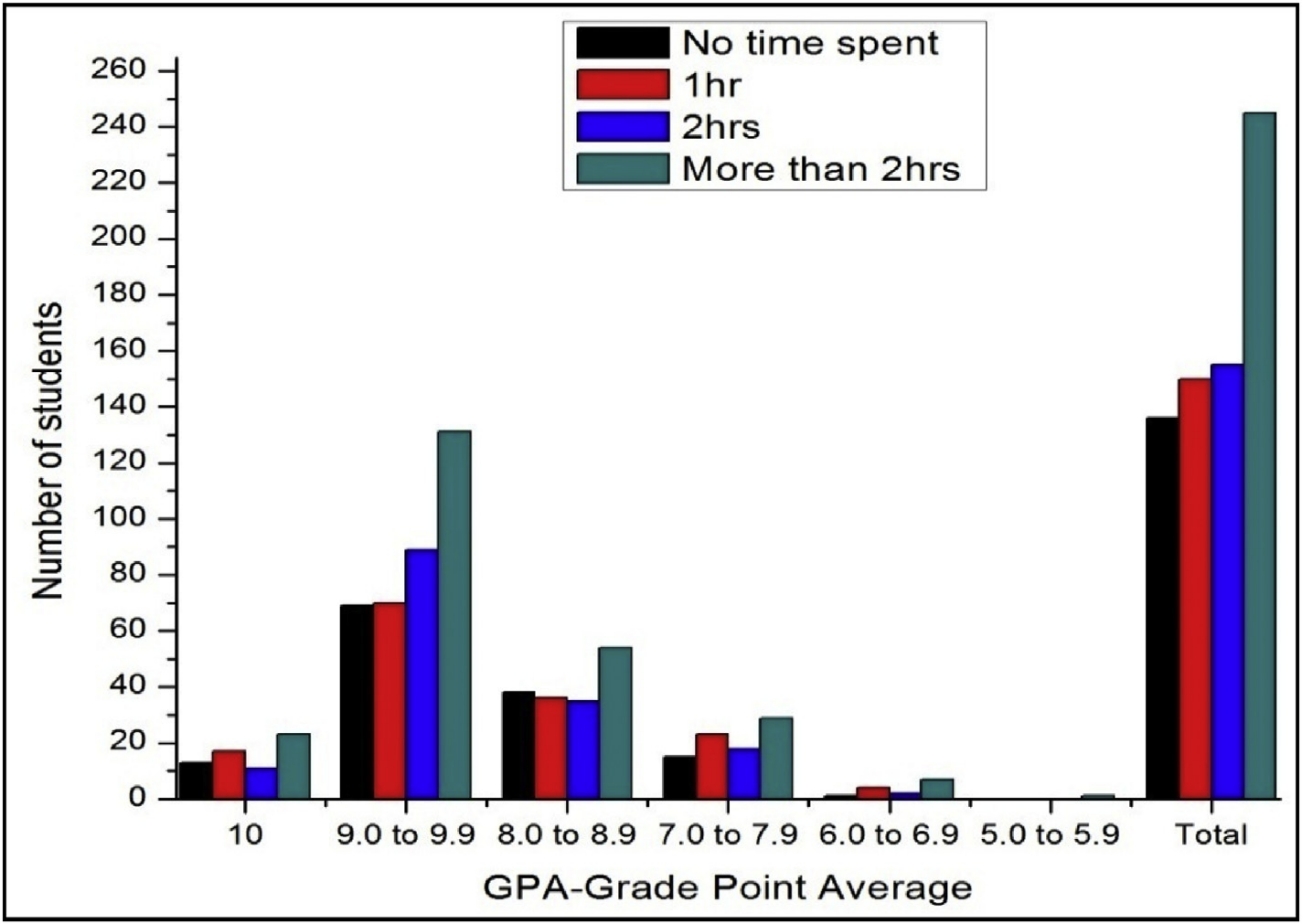
The bar chart shows the number of male students' GPA (the academic year 2017–18) based time spent on study after school per day.

The total number of female and male students’ GPA based on time spent for study after school shown in Fig. 7, Fig. 8.

**Fig. 9:**
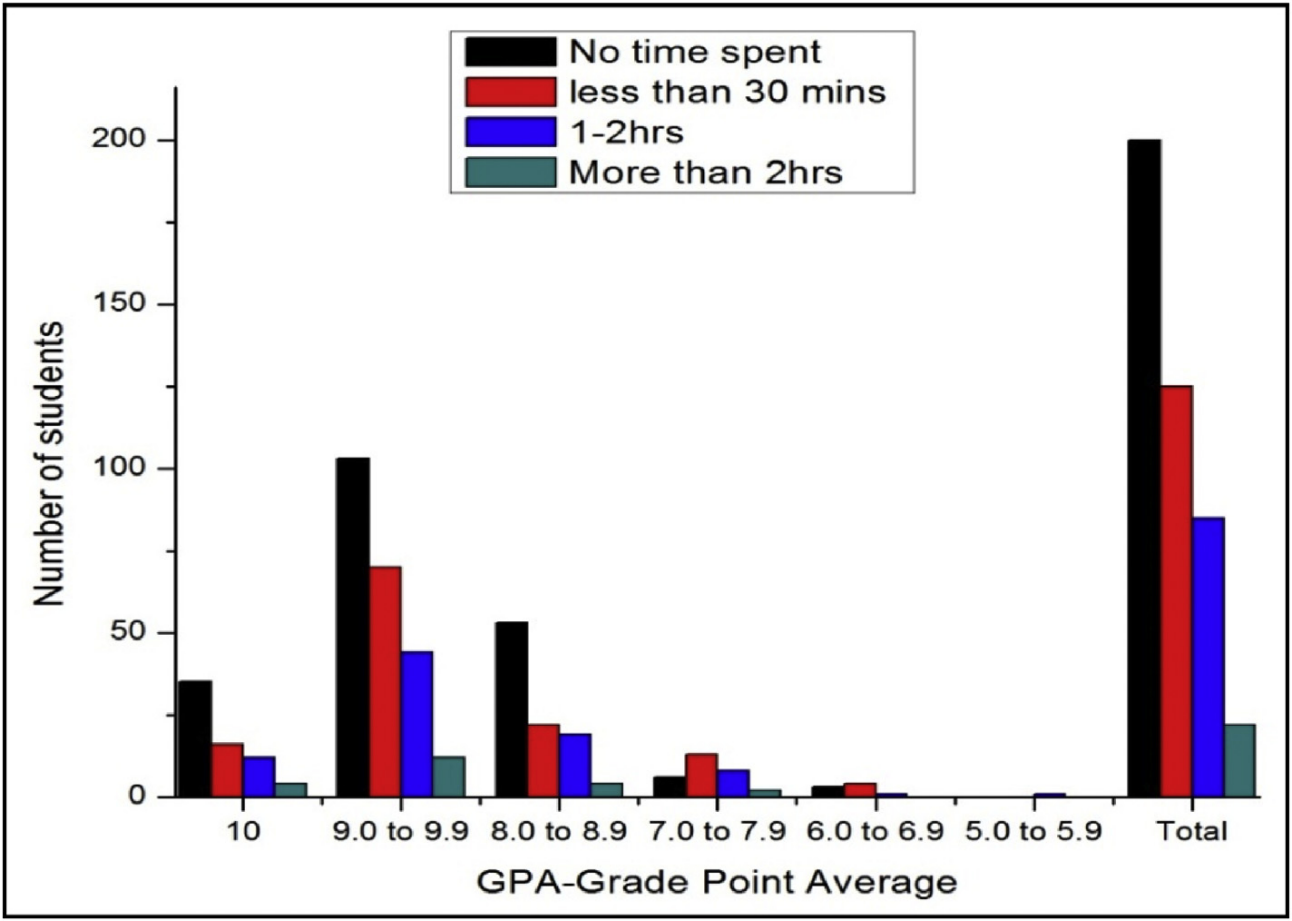
The bar chart shows the number of female students' GPA (the academic year 2017–18) based time spent on sports per day.

**Fig. 10:**
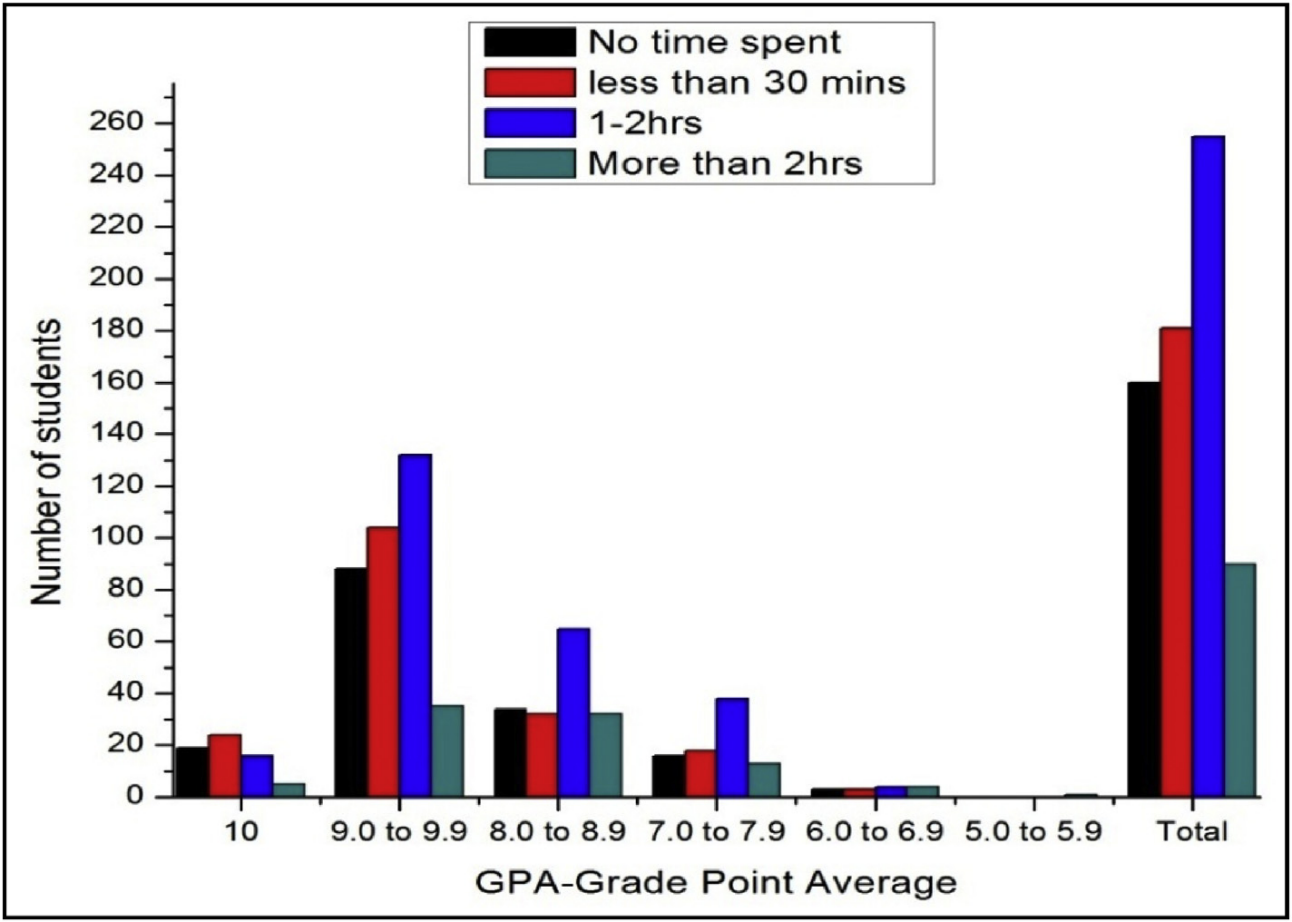
The bar chart shows the number of male students' GPA (the academic year 2017–18) based time spent on sports per day.

The total number of female and male students’ GPA based on time spent on sports shown in Fig. 9, Fig. 10.

**Fig. 11:**
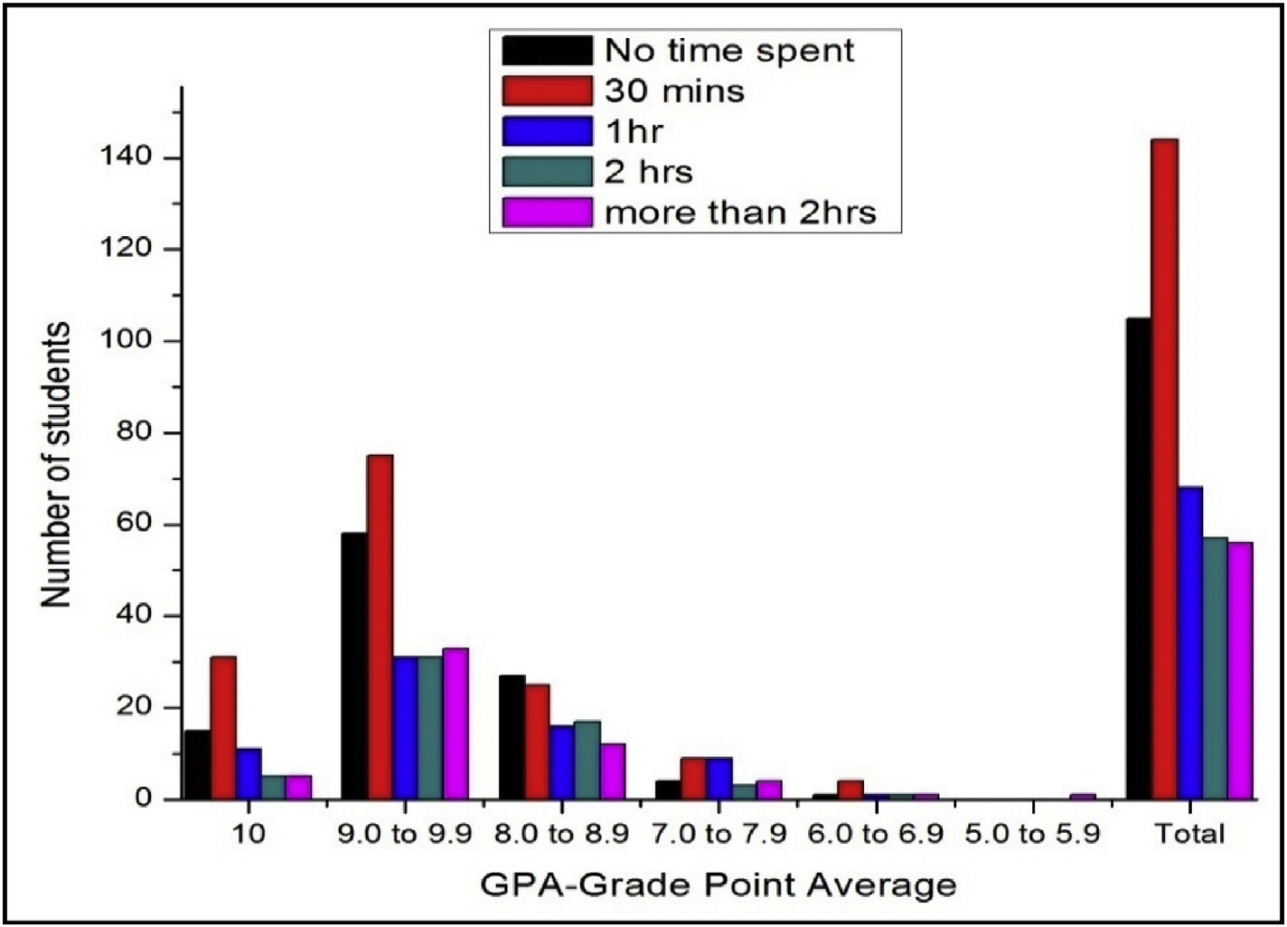
The bar chart shows the number of female students' GPA (the academic year 2017–18) based time spent with mobile per day.

**Fig. 12:**
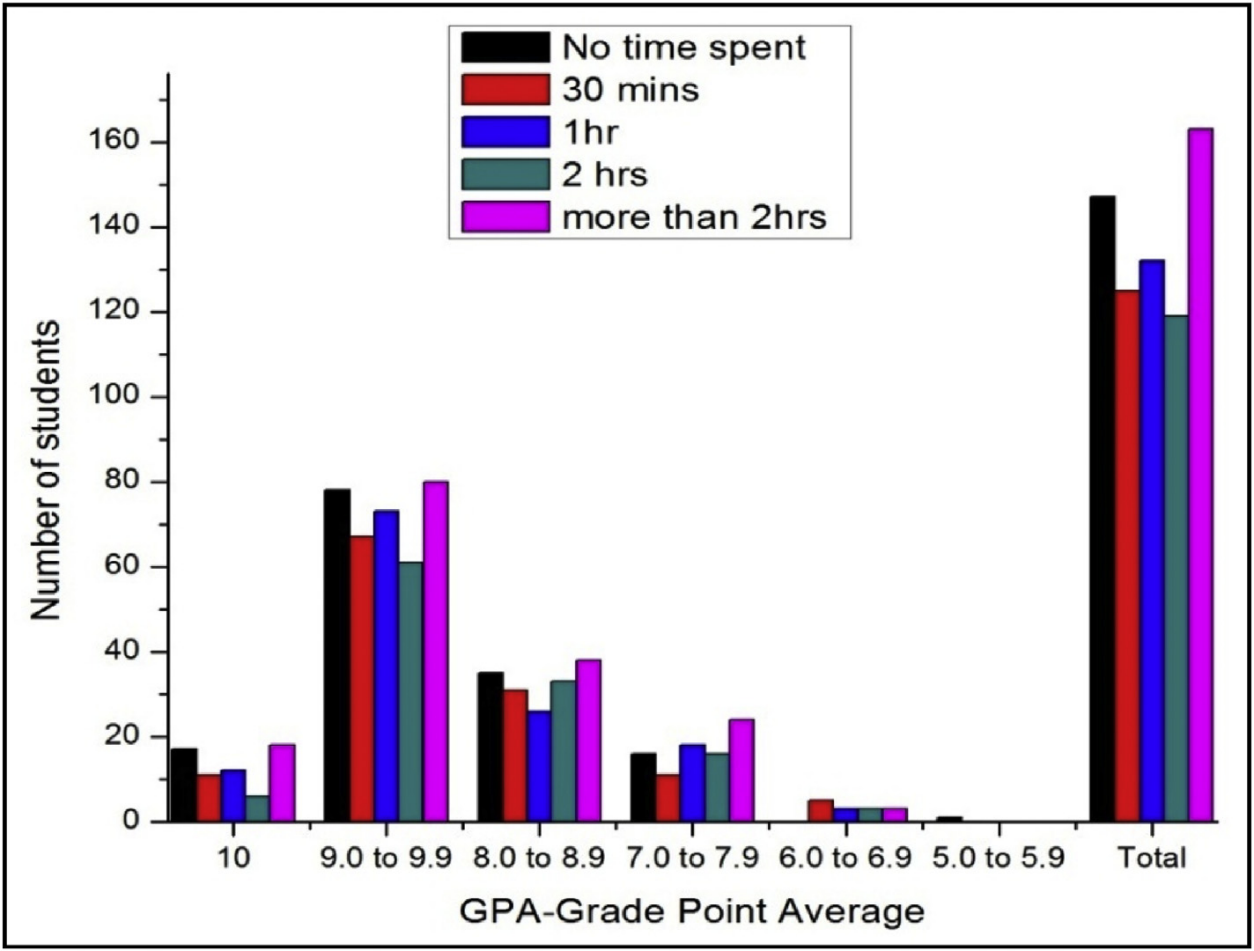
The bar chart shows the number of male student's GPA (the academic year 2017–18) based time spent with mobile per day.

The total number of female and male student's GPA based on time spent with mobile per day shown in Fig. 11, Fig. 12.

**Fig. 13:**
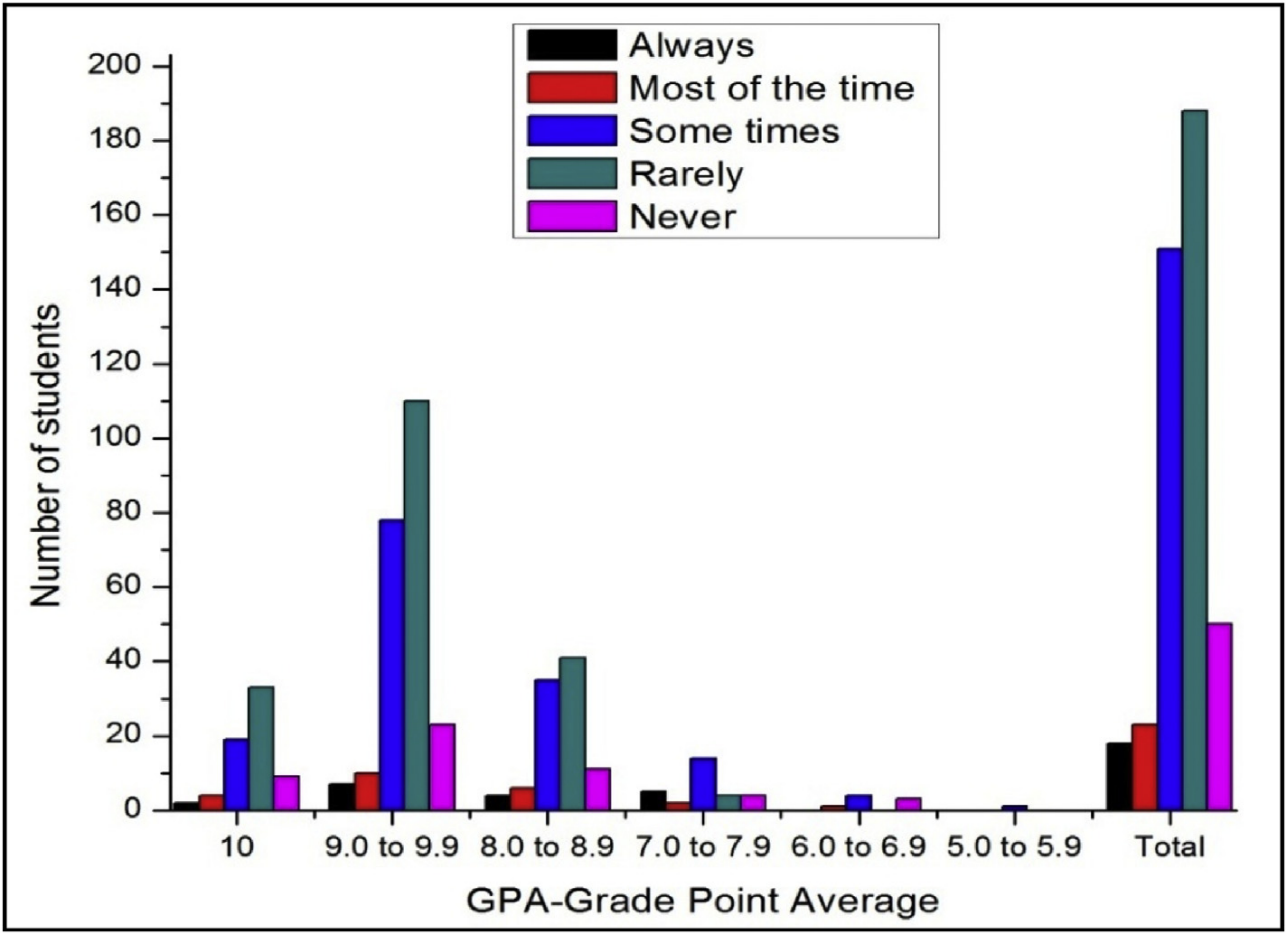
The bar chart shows the number of female students' GPA (the academic year 2017–18) based impact of health problems.

**Fig. 14:**
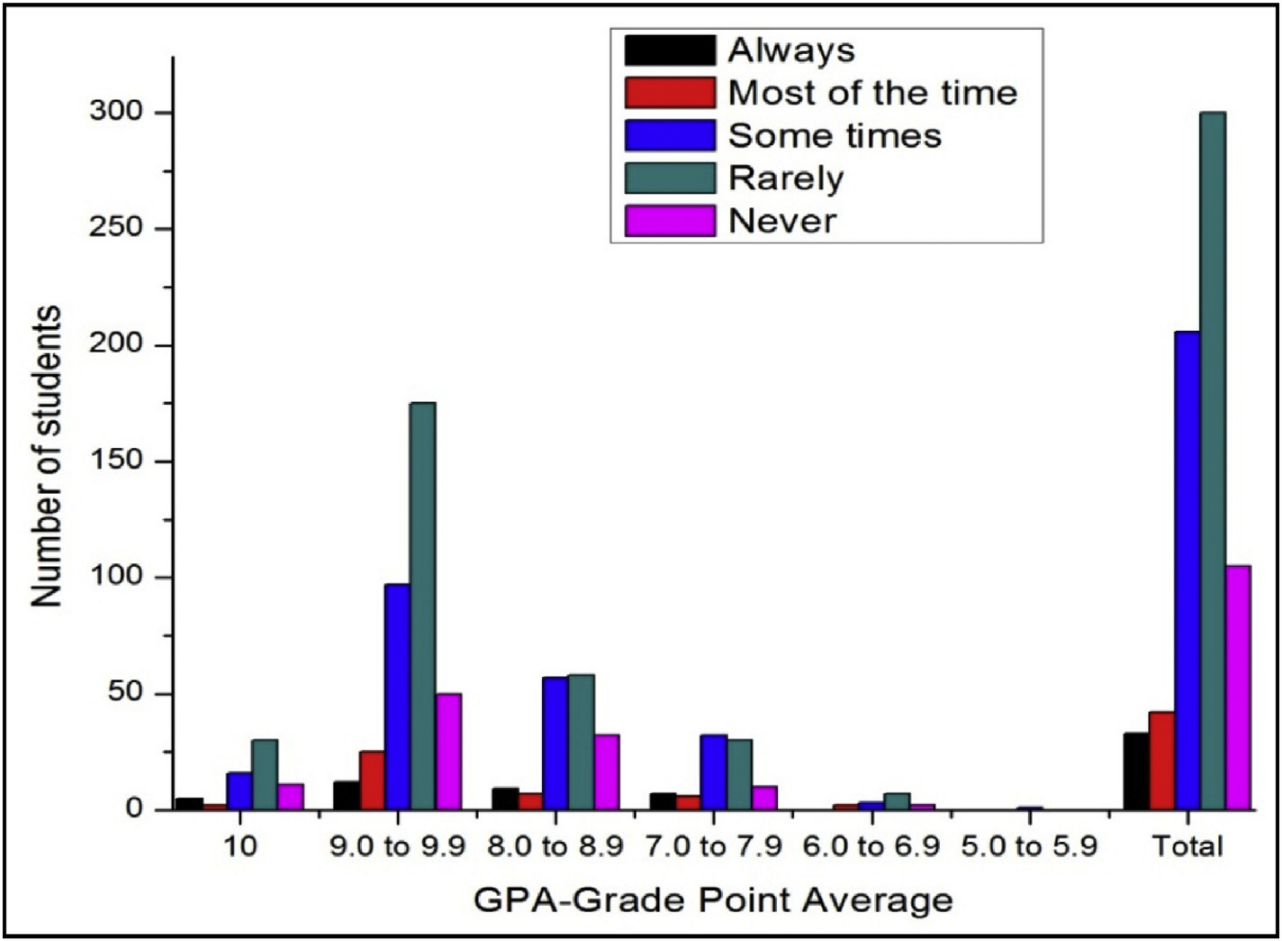
The bar chart shows the number of male students' GPA (the academic year 2017–18) based impact of health problems.

The total number of female and male students’ GPA based on the impact of health problems shown in Fig. 13, Fig. 14.

**Fig. 15:**
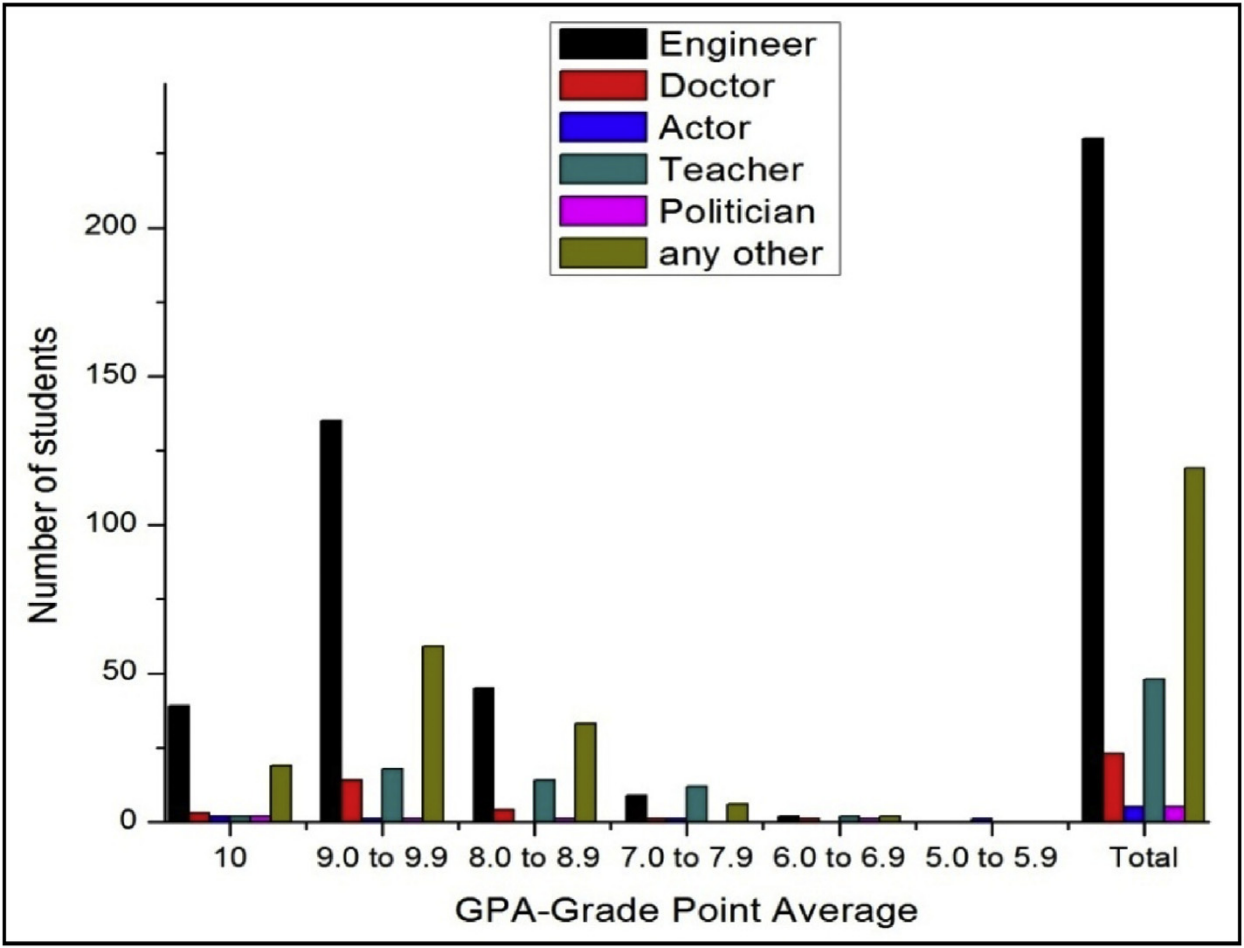
The bar chart shows the number of female students' GPA (the academic year 2017–18) based impact of goal.

**Fig. 16:**
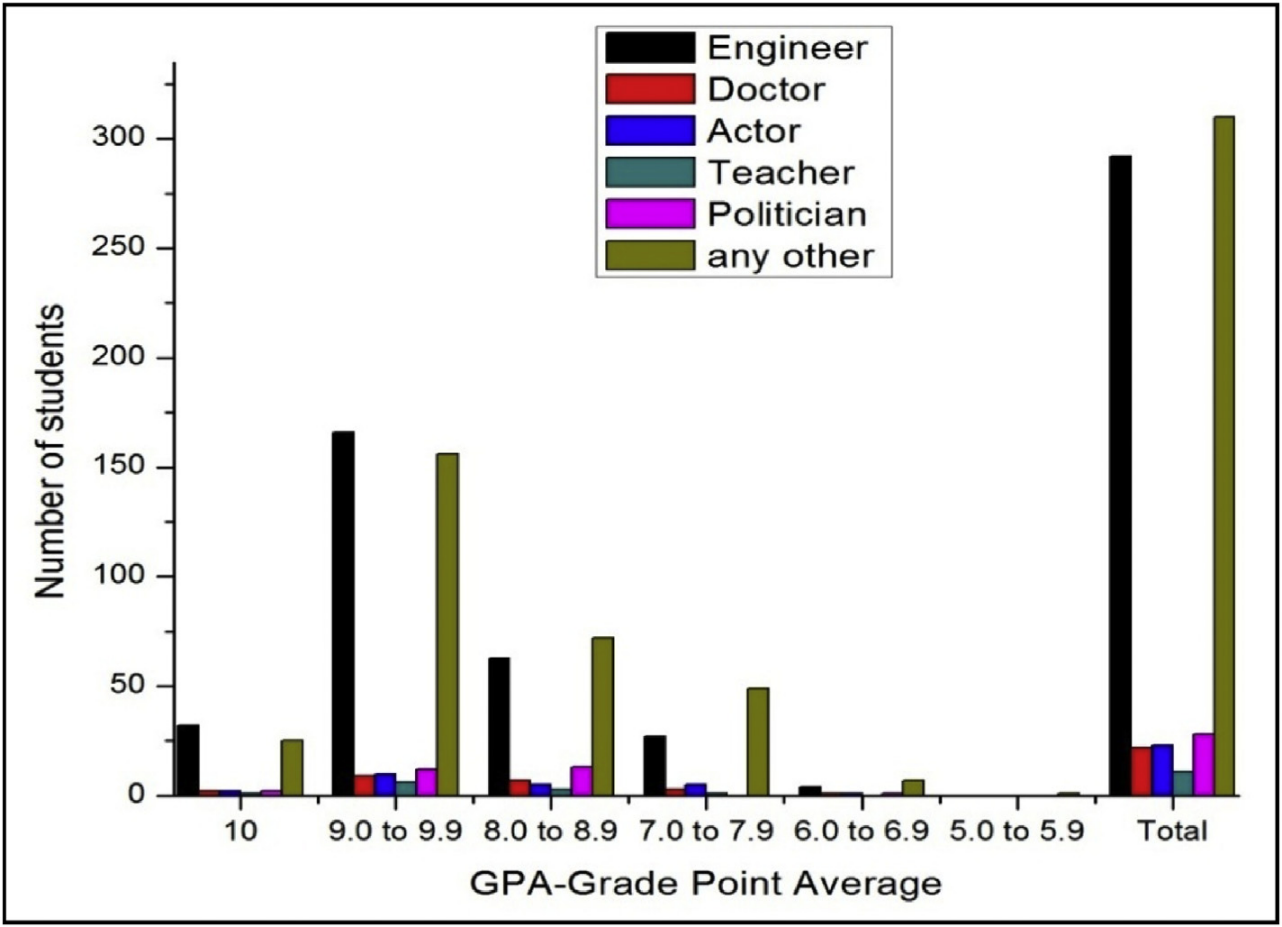
The bar chart shows the number of male students' GPA (the academic year 2017–18) based impact of goal.

The total number of female and male students’ GPA based on the goal shown in Fig. 15, Fig. 16.

**Fig. 17:**
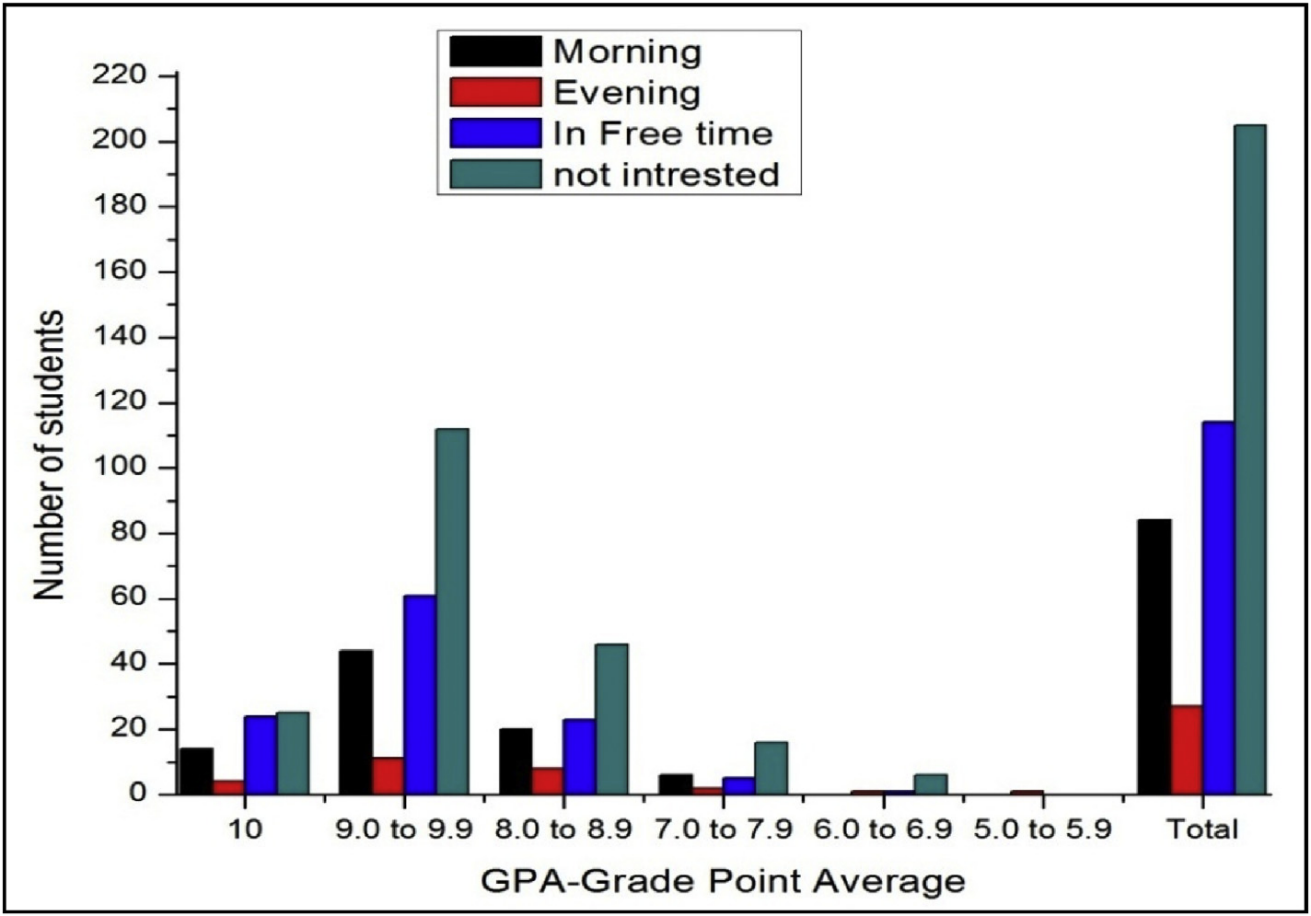
The bar chart shows the number of female students' GPA (the academic year 2017–18) based time spent on yoga or physical exercise.

**Fig. 18:**
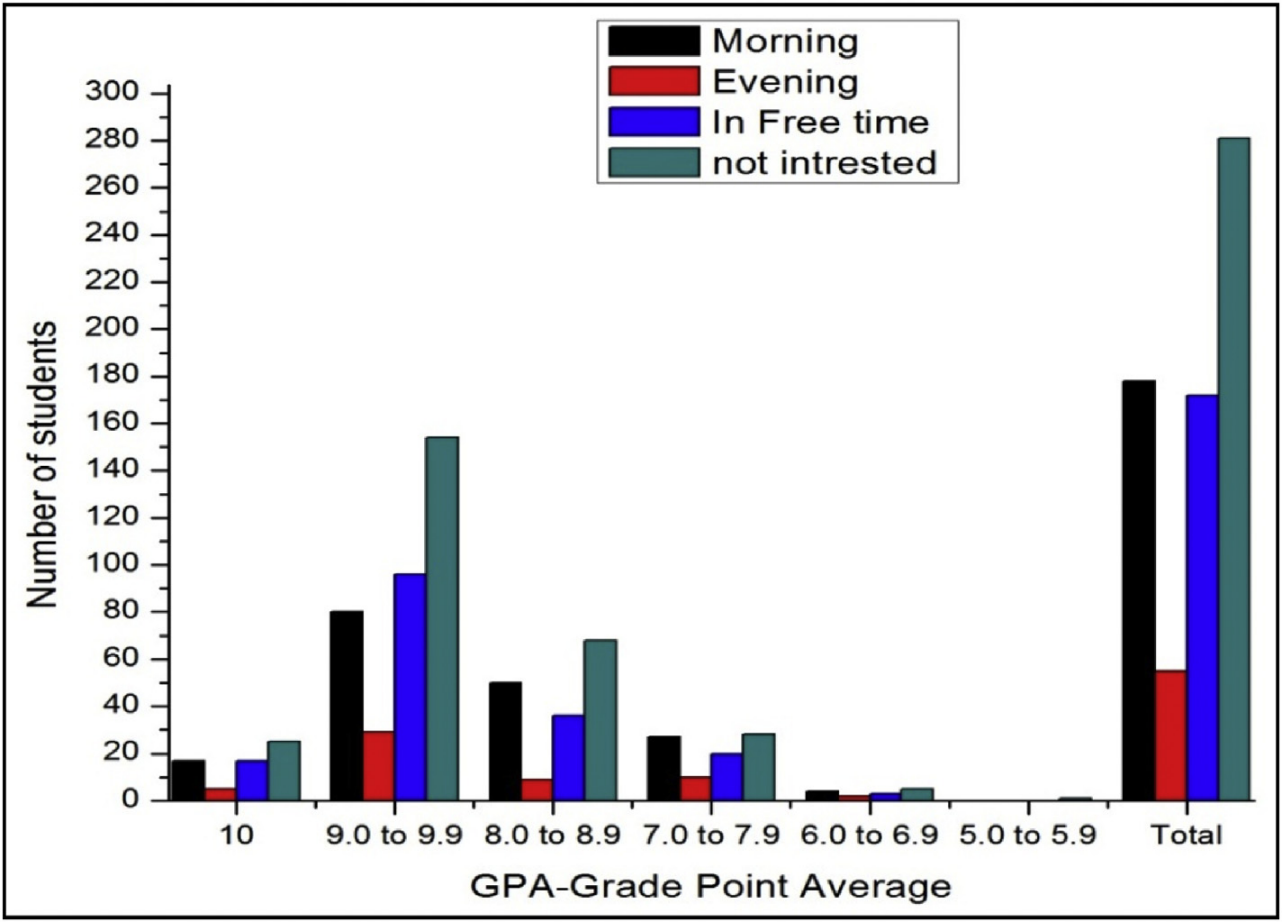
The bar chart shows the number of male students' GPA (the academic year 2017–18) based time spent on yoga or physical exercise.

The total number of female and male student's GPA based on time spent on yoga or physical exercise shown in Table 21, Table 22, Table 23, Table 24, Table 25, Table 26, Table 27 and Fig. 17, Fig. 18.

**Table 21:** The number of male students’ GPA (the academic year 2017–18) based time spent with mobile per day.

GPA	No time spent	30 mins	1hr	2 hrs	More than 2hr
10	17	11	12	6	18
9.0 to 9.9	78	67	73	61	80
8.0 to 8.9	35	31	26	33	38
7.0 to 7.9	16	11	18	16	24
6.0 to 6.9	0	5	3	3	3
5.0 to 5.9	1	0	0	0	0
Total	147	125	132	119	163

**Table 22:** The number of female students’ GPA (the academic year 2017–18) based impact of health problems.

GPA	Always	Most of the time	Some times	Rarely	Never
10	2	4	19	33	9
9.0 to 9.9	7	10	78	110	23
8.0 to 8.9	4	6	35	41	11
7.0 to 7.9	5	2	14	4	4
6.0 to 6.9	0	1	4	0	3
5.0 to 5.9	0	0	1	0	0
Total	18	23	151	188	50

**Table 23:** The number of male students’ GPA (the academic year 2017–18) based impact of health problems.

GPA	Always	Most of the time	Some times	Rarely	Never
10	5	2	16	30	11
9.0 to 9.9	12	25	97	175	50
8.0 to 8.9	9	7	57	58	32
7.0 to 7.9	7	6	32	30	10
6.0 to 6.9	0	2	3	7	2
5.0 to 5.9	0	0	1	0	0
Total	33	42	206	300	105

**Table 24:** The number of female students’ GPA (the academic year 2017–18) based impact of goal.

GPA	Engineer	Doctor	Actor	Teacher	Politician	any other
10	39	3	2	2	2	19
9.0 to 9.9	135	14	1	18	1	59
8.0 to 8.9	45	4	0	14	1	33
7.0 to 7.9	9	1	1	12	0	6
6.0 to 6.9	2	1	0	2	1	2
5.0 to 5.9	0	0	1	0	0	0
Total	230	23	5	48	5	119

**Table 25:** The number of male students’ GPA (the academic year 2017–18) based impact of goal.

GPA	Engineer	Doctor	Actor	Teacher	Politician	any other
10	32	2	2	1	2	25
9.0 to 9.9	166	9	10	6	12	156
8.0 to 8.9	63	7	5	3	13	72
7.0 to 7.9	27	3	5	1	0	49
6.0 to 6.9	4	1	1	0	1	7
5.0 to 5.9	0	0	0	0	0	1
Total	292	22	23	11	28	310

**Table 26:** The number of female students’ GPA (the academic year 2017–18) based time spent on yoga or physical exercise.

GPA	Morning	Evening	In Free time	not interested
10	14	4	24	25
9.0 to 9.9	44	11	61	112
8.0 to 8.9	20	8	23	46
7.0 to 7.9	6	2	5	16
6.0 to 6.9	0	1	1	6
5.0 to 5.9	0	1	0	0
Total	84	27	114	205

**Table 27:** The number of male students’ GPA (the academic year 2017–18) based time spent on yoga or physical exercise.

GPA	Morning	Evening	In Free time	not interested
10	17	5	17	25
9.0 to 9.9	80	29	96	154
8.0 to 8.9	50	9	36	68
7.0 to 7.9	27	10	20	28
6.0 to 6.9	4	2	3	5
5.0 to 5.9	0	0	0	1
Total	178	55	172	281
